# Outcome of no oral antibiotic prophylaxis and bowel preparation in Crohn’s diseases surgery

**DOI:** 10.1007/s00508-019-1475-8

**Published:** 2019-03-06

**Authors:** Lukas Walter Unger, Stefan Riss, Stanislaus Argeny, Michael Bergmann, Thomas Bachleitner-Hofmann, Friedrich Herbst, Anton Stift

**Affiliations:** 1 Division of General Surgery, Department of Surgery, Medical University of Vienna, Währinger Gürtel 18–20, 1090 Vienna, Austria; 2 Department of Surgery, Hospital Barmherzige Brüder, 1020 Vienna, Austria

**Keywords:** Inflammatory bowel disease, Minimally invasive surgery, Infectious complication, Fast track, Mechanical bowel preparation

## Abstract

**Background:**

Recent studies support the use of mechanical bowel preparation and/or oral antibiotic prophylaxis in patients operated on for Crohn’s disease (CD); however, data are scarce, especially for laparoscopic surgery. Therefore, this study was carried out to investigate the effect of laparoscopic surgery on complication rates in patients not undergoing standardized bowel preparation but single shot antibiotics.

**Methods:**

In this study 255 consecutive patients who underwent a laparoscopic intestinal resection for CD at a tertiary referral center between 1997 and 2014 were retrospectively analyzed. Superficial surgical site infections (SSI), organ/space infections and ileus were recorded and grouped according to the type of resection (colorectal vs. small intestine ± ileocecal).

**Results:**

The baseline characteristics of the groups were comparable. Colorectal resections showed a significantly increased risk of organ/space infection (4.6% in small intestine ± ileocecal vs. 14.3% in colorectal resections *p* = 0.039). The superficial SSI rate was low in both groups (1.8% in small intestine ± ileocecal resection vs. 0% in colorectal resections, *p* = 1.000). Univariate binary logistic regression analysis revealed a statistically significant influence of duration of surgery (*p* = 0.001) and type of resection (*p* = 0.031) on organ/space infection. In multivariate analysis, only duration of surgery (OR 1.111, 95% CI 1.026–1.203 for every 10 min, *p* = 0.009) remained significant for postoperative organ/space infections.

**Conclusions:**

Single-shot antibiotic therapy without bowel preparation is safe in patients undergoing minimally invasive surgery and was associated with a low number of complications; however, organ/space infections were more common if colorectal resections were performed. Therefore, combined bowel preparation might be beneficial when the (sigmoid) colon or rectum are involved.

## Introduction

There is an ongoing debate among colorectal surgeons about the use of bowel preparation and oral administration of non-absorbable antimicrobial agents prior to surgery, despite clear recommendations by recent guidelines [1]. Although investigators in the past did not find any beneficial effects [2], recent evidence from large studies has shown that combined bowel preparation can significantly reduce surgical site infections (SSI) and anastomotic leak in elective (open) colorectal resections [3, 4]. In contrast, other randomized controlled studies have even suggested negative effects of bowel preparation with increased morbidity and electrolyte changes [5, 6]. Notably, there is less evidence for bowel preparation in inflammatory bowel disease (IBD), especially for Crohn’s disease (CD). As patients with CD are often malnourished and commonly present with intraoperative abscesses, strictures, fistulas and perforations at the time of surgery, SSI commonly occur postoperatively [7, 8]. Moreover, the majority of patients suffer from diarrhea and significant stenosis at the time of presentation [9], thus mechanical bowel preparation may be contraindicated. These assumptions are in line with a large recently published study that did not find any additional benefit in mechanical preparation compared to antibiotic prophylaxis alone the day before colorectal surgery [10]. Although evidence is scarce, a recent randomized controlled trial found that combined use significantly reduced SSI in open surgery for CD [11]. Due to the special nature of CD and risk of aspiration, in the author’s center bowel preparation is not carried out before surgery for CD. In addition, laparoscopy is conducted whenever possible and has proven to be safe, even in penetrating CD [12]. This potentially leads to a significantly reduced rate of SSI irrespective of additional bowel preparation. Nevertheless, the affected area (namely small vs. large intestine) rather than the type of disease might be of importance regarding SSI and organ space infections. Due to the lack of data the present study was conducted to assess the safety of no bowel preparation on the outcome of laparoscopic surgery in CD and identify potential subgroups that might benefit from combined bowel preparation in the future.

## Material and methods

### Setting and participants

This study was designed as a retrospective cohort study. Consecutive patients (*n* = 255) who underwent intestinal resection for CD by a single colorectal surgical team at a tertiary referral center between 1997 and 2014 were included. The surgical technique, in particular the laparoscopic approach, has already been described in detail [13].

### Single shot antibiotic therapy

Single shot antibiotic therapy (consisting of cefuroxime and metronidazole) is administered 30 min before skin incision. In the case of allergies, individual adaptation of the antibiotic regimen is performed. In the case of kidney impairment, the dose is adapted as given by the manufacturer’s enclosed instructions. In cases of intraoperatively identified intra-abdominal abscesses, antibiotic therapy is prolonged to postoperative day 5 and is adapted when the antibiogram is available.

### Variables

Baseline characteristics such as age, body mass index (BMI), sex, smoking status at the time of surgery, the use of immunosuppressive medication within 1 week prior to surgery and the number of previous abdominal operations were recorded. To assess the perioperative outcome, intra-abdominal findings (e.g. stenosis, abscess, conglomerate tumor, fistula), urgency of surgery (emergency vs. elective), operative time, complications (Clavien-Dindo classification [14]) and length of hospital stay (days) were analyzed. When conversion was necessary, the patient was still analyzed as laparoscopic surgery (intention to treat). Conversion was defined as the incision length exceeding 6 cm. The SSIs were defined in accordance with the Center for Disease Control definition [15]. In brief, an infection was defined when any of the following occurred: purulent discharge from the incision of the placed drainage into the organ/space, detection of organisms by culturing fluid/tissue from the incision or organ space/drainage, clinical signs of infection of an open wound and detection of an abscess or other evidence of infection in the incision/organ space, including computed tomography (CT) scans. None of the included patients had received mechanical or oral antibiotic bowel preparation. Follow-up was performed in the outpatient clinic and SSI and complications were assessed within a 30-day period following surgery. All variables were collected during routine clinical work. Data were recorded prospectively but retrospectively analyzed.

### Ethical considerations

The retrospective study was approved by the Medical University of Vienna review board (EK 1837/2017; available at https://ekmeduniwien.at/core/catalog/2017/). The study was conducted in accordance with the STROBE guidelines [16].

### Statistical analysis

Continuous variables are presented as median (interquartile range). As normal distribution could not be assumed after Kolmogorov Smirnov testing, the Mann Whitney U-test was used to compare between groups in continuous variables. For categorical variables, 2‑sided Fisher’s exact test was used to compare groups. For univariate and multivariate analysis of superficial surgical site infection, organ/space infection, ileus or leak, a binary logistic regression analysis of variance was calculated. *P*-values <0.25 in the univariate analysis were used for multivariate analysis. The SPSS version 24 (IBM, Armonk, NY, USA) was used for statistical analysis. A *p*-value of *p* < 0.05 was considered to denote statistical significance.

## Results

Baseline characteristics are shown in Table 1. Baseline characteristics of patients undergoing small intestinal resections and/or ileocecal resection (ICR) were comparable to patients undergoing colonic and/or rectal resections. Importantly, however, patients with colonic and/or rectal resections had a higher white blood cell count (WBC) (7.6 G/L, IQR 4.3 vs. 10.4 G/L, IQR 7.6, *p* = 0.011) although undergoing surgery while on active immunosuppression more often (29.1% vs. 45.7%, respectively, *p* = 0.049).

**Table 1:** Baseline characteristics of Crohn’s disease patients according to each group (small intestine and/or ICR vs. colon and/or rectal resections)

	Small intestinal resections and/or ICR (*n* = 220)	Colon or rectal resection (*n* = 35)	*p*-value
Age (years)	32.0 (15.8)	32.5 (24.3)	0.402
Female gender (%)	98 (38.4)	17 (48.6)	0.716
BMI (kg/m^2^)	21.4 (4.7)	21.4 (5.7)	0.921
Active smoker (%)	105 (47.7)	17 (48.6)	1.000
Hemoglobin (g/dL)	12.5 (2)	12.0 (3)	0.277
WBC (G/L)	7.6 (4.3)	10.4 (7.6)	***0.011***
CRP (mg/dL)	1.38 (4)	1.99 (6)	0.275
Albumin (mg/dL)	38.5 (7.3)	36.0 (7.7)	0.075
Perioperative immunosuppressive medication (%)	64 (29.1)	16 (45.7)	***0.049***
Prior abdominal operations			
None (%)	173 (78.6)	34 (97.1)	0.067
1 (%)	31 (14.1)	0 (0)	
2 (%)	12 (5.5)	1 (2.9)	
≥3 (%)	4 (1.8)	0 (0)	

Continuous variables given as median and interquartile range (IQR)

*BMI* body mass index, *CRP* C-reactive protein; *ICR* ileocecal resection, *WBC* white blood cell count

### Intraoperative findings and postoperative outcome

Regarding the complexity of surgery and CD activity, no differences were observed between the two groups. The most commonly observed pathology was stenosis (79.1% vs. 82.9%, *p* = 0.821 in the small intestines and/or ICR vs. colon and/or rectal resections), followed by fistulas (49.1% vs. 57.1%, *p* = 0.467). All operations were intended as laparoscopic resections; however, conversion rates were 15.5% and 22.9% (*p* = 0.324), respectively. The operation time had a tendency towards longer duration in colon and/or rectal resections (*p* = 0.068) but differences did not reach statistical significance. The median length of in-hospital stay was comparable between groups (8 (IQR 3) vs. 9 (IQR 6) days, *p* = 0.158). Superficial surgical site infections were an absolute rarity. Nevertheless, organ/space infections were more common in the colon or rectal resection group (4.6% vs. 14.3%; *p* = 0.039), while ileus rates were comparable (5.9% vs. 8.6%; *p* = 0.467).

The findings are presented in Table 2.

**Table 2:** Procedural characteristics and postoperative outcome of Crohn’s disease patients according to each group (small intestine and/or ICR vs colon and/or rectal resections)

	Small intestinal resections and/or ICR (*n* = 220)	Colon or rectal resection (*n* = 35)	*p*-value
*Intraoperative findings*			
Stenosis (%)	174 (79.1)	29 (82.9)	0.821
Abscess (%)	44 (20.0)	4 (11.4)	0.350
Conglomerate tumor (%)	91 (41.4)	15 (42.9)	0.856
Fistula (%)	108 (49.1)	20 (57.1)	0.467
*Conversion (%)*	34 (15.5)	8 (22.9)	0.324
*Emergency operation (%)*	8 (3.6)	3 (8.6)	0.180
*Operative time (min)*	135 (75)	162 (116)	0.068
*Length of hospital stay (days)*	8 (3)	9 (6)	0.158
*Superficial SSI (%)*	4 (1.8)	0 (0)	1.000
*Organ/space infection (%)*	10 (4.6)	5 (14.3)	**0.039**
*Ileus (%)*	13 (5.9)	3 (8.6)	0.467

Continuous variables given as median (IQR)

*ICR* ileocecal resection, *SSI* surgical site infection

### Univariate and multivariate analysis on organ space infections

To assess the influence of operative time, sex, active immunosuppression, intraoperative findings, BMI, type of resection and laboratory values as well as number of prior surgeries and conversion, a univariate binary logistic regression analysis was conducted. Interestingly, the duration of surgery, active immunosuppression, type of resection and conversion showed either a significant influence or a trend towards an increased risk for organ space infections and were therefore selected for multivariate analysis. In multivariate analysis, only duration of surgery (OR 1.111, 95% CI 1.026–1.203 for every 10 min, *p* = 0.009) showed a statistically significant influence on postoperative organ space infection. Colon and/or rectal resections (*p* = 0.101) as well as necessary conversion (*p* = 0.145) showed a trend towards increased risk of organ space infection.

Results are presented as Table 3.

**Table 3:** Univariate and multivariate binary logistic regression analysis of variance on occurrence of organ space infection

	Univariate analysis				Multivariate analysis			
–	OR	95%CI		*p* value	OR	95%CI		*p* value
		Lower	Upper			Lower	Upper	
Duration of surgery (for every 10 min)	1.132	1.050	1.20	***0.001***	1.111	1.026	1.203	***0.009***
Sex (male vs. female)	0.591	0.196	1.781	0.350	–	–	–	–
Immunosuppression at the time of surgery (vs. no immunosuppression)	0.512	0.179	1.464	0.212	1.709	0.514	5.681	0.382
Stenosis vs. no stenosis	0.688	0.210	2.254	0.536	–	–	–	–
Fistula vs. no fistula	0.802	0.308	2.489	0.802	–	–	–	–
Conglomerate tumor vs. no tumor	0.688	0.228	2.075	0.507	–	–	–	–
BMI (for every kg/m^2^)	0.997	0.872	1.140	0.965	–	–	–	–
Colon and/or rectal resection vs. small bowel and/or ICR	3.500	1.120	10.940	***0.031***	2.882	0.814	10.207	0.101
Albumin pre-OP (for every mg/dL)	1.058	0.945	1.185	0.328	–	–	–	–
WBC pre-OP (every G/L)	0.996	0.854	1.162	0.963	–	–	–	–
Conversion vs. no conversion	2.743	0.887	8.486	0.080	2.506	0.729	8.609	0.145
Re-OP vs. index-OP	1.083	0.293	3.999	0.904	–	–	–	–

*BMI* body mass index, *pre-OP* preoperatively, *index-OP* indexoperation, *re-OP* reoperation, *WBC* white blood cell count, *ICR* ileocecal resection, *OR* odds ratio, *CI* confidence interval

## Discussion

In the present study a low number of complications could clearly be demonstrated in patients operated on laparoscopically for symptomatic CD without using preoperative mechanical bowel preparation but single shot antibiotics only. As a consequence, bowel preparation before surgery is not essential for an uncomplicated postoperative course in this specific group of patients, assuming that single shot antibiotics is repeated after 120 min of operative time.

In the literature there is an increasing body of evidence that combined bowel preparation is useful in left-sided colon and rectal resection. Nichols et al. published the effectiveness of additional oral antibiotics more than 40 years ago [17]; however, the topic of optimal preparation in (elective) surgery still remains of interest [3, 18, 19]. In the study by Ohman et al. an infection prevention bundle consisting of preoperative oral antibiotics, mechanical bowel preparation and shower with chlorhexidine containing cleanser and antibiotic irrigation in addition to a clean closure protocol intraoperatively led to a significant reduction of SSI from 21.2% in 2011 to 6.0% in 2015 [19]; however, the study did not differentiate between laparoscopy and open surgery and type of procedure. Nevertheless, the anatomical location of the disease, indications for surgery and potential confounding factors, such as preoperative stenosis, need to be taken into account to accurately define the influence of preparation prior to surgery. Vo et al. [3] reported a more pronounced decrease of SSI in left colonic or rectal surgery compared to right-sided hemicolectomy when patients without preoperative oral antibiotics were compared to patients with combined preparation. This is in line with the findings of a large retrospective analysis of the National Surgical Quality Improvement Program database that revealed a decreased rate of anastomotic leakage after right hemicolectomy as well as a reduced rate following laparoscopic partial colectomy compared to the entire population analyzed [20]. In another analysis of the ACS-NSQIP database by Klinger et al. [21], combined preparation was superior to either mechanical or antibiotic preparation alone; however, the etiology of the underlying disease was not reported. Notably, the majority of recently published studies are of a retrospective nature [3, 10, 22] and several prospective randomized studies have not shown any beneficial effect [2, 5, 23]. Moreover, patient characteristics and pathophysiology differ in CD compared to colorectal malignancies. In an ACS-NSQIP study focusing on IBD, however, a statistically significant benefit in combined preparation compared to single preparation or no preparation was found [24]. Notably, most patients in the study (42.5%) did not receive any bowel preparation, indicating that there is no clear consensus on optimal preoperative therapy in IBD. Moreover, ulcerative colitis as well as CD are combined and not separately evaluated. Finally, the authors did not differentiate between laparoscopic and open approaches. In contrast to their results, the present analyses on laparoscopically (started or performed) resections revealed an overall incisional SSI rate of 1.6% which is lower than reported by other authors. Therefore, especially in laparoscopic ileocecal and small intestinal resection, the role of bowel preparation needs to be evaluated in prospective, controlled multicenter studies and stratified for preoperative risk factors. In line with this center’s standard of avoiding mechanical bowel preparation in CD patients, a review by Zangenberg et al. evaluated preoperative optimization strategies and concluded that “the evidence to support bowel preparation is not well established” and recommend avoiding bowel preparation until further data from ongoing studies are available [25]. In a recently published study by Iesalnieks et al. [26] on the other hand, mechanical bowel preparation significantly reduced intra-abdominal septic complications. Importantly, stenosis rates were lower in their cohort (27% structuring disease) and only 14% underwent laparoscopic surgery. As a conclusion, the authors recommend mechanical bowel preparation (MBP) in (open) colon resections. As their rate of preoperative oral antibiotics was low, however, no definitive conclusion on a combined regimen can be drawn. This is of interest as oral antibiotic therapy in addition to MBP has been shown to reduce the number of incisional SSI in open proctocolectomy procedures [27], however, the referenced study only included colitis patients who received additional oral antibiotics in addition to MBP. In favor of avoiding bowel preparation and implementing minimally invasive surgery whenever possible, an enhanced recovery pathway without MBP in laparoscopic CD surgery did not lead to increased complication rates with shorter hospital stay in a study by Spinelli et al. [28]. Notably, SSI per se were not reported in the study but Clavien-Dindo grade I–II complications (which included opening of a wound at the bedside) were similar.

In open surgery for CD, MBP with picosulfate hydrate combined with three doses of kanamycin and metronidazole the day before surgery and intravenous second generation cephalosporins 30 min before surgery with prolonged administration for 24 h was shown to significantly decrease incisional SSI [11]. Interestingly, the rates of SSI were relatively high in the cited study and the most commonly performed procedure was colonic resection in the respective subgroups, thereby representing significant differences in the patient population compared to the present study. Due to the high incidence of stenosis in the present study and the significant difference in organ space infections between small bowel/ICR and colon/rectal resections, oral antibiotics might show favorable results; however, MBP should be used with caution, leading to potential aspiration risks. Importantly, however, few limitations of the present study need to be addressed. Although a large number of consecutive patients who were operated on for CD were included, patients were not randomized, thus selection bias cannot be ruled out. Due to a clear and constant perioperative regimen, data are well comparable between groups and between other study cohorts; however, it is worth mentioning that patients undergoing colon/rectal resections were more commonly under active immunosuppression with either corticosteroids or azathioprine and had a higher WBC, being a potential surrogate parameter for dwelling infections, although the intraoperative rate of detected abscesses was similar and neither WBC nor immunosuppression showed a significant influence on organ space infections in univariate or multivariate analysis. Interestingly, however, the type of resection showed a statistically significant influence in univariate but not multivariate analysis. These findings underline the importance of future prospective, well-designed multicenter studies accounting for center differences in hygiene standards. Importantly, a control group with oral antibiotics but without MBP should be added as diarrhea and stenosis might obviate the need for active MBP.

In conclusion, laparoscopic surgery for CD is safe and associated with a low number of complications without using mechanical and antibiotic bowel preparation. Bowel preparation may have beneficial effects in colon resection, which needs to be addressed by future prospective trials.

## Abbreviations

BMI: Body mass index
CI: Confidence interval
CRP: C-reactive protein
CT: Computed tomography
IBD: Inflammatory bowel disease
ICR: Ileocecal resection
IQR: Interquartile range
MBP: Mechanical bowel preparation
OR: Odds ratio
SSI: Surgical site infection
WBC: White blood cell count

## References

[1] Carmichael JC, Keller DS, Baldini G, Bordeianou L, Weiss E, Lee L (2017). Clinical practice guidelines for enhanced recovery after colon and rectal surgery from the American Society of Colon and Rectal Surgeons and Society of American Gastrointestinal and Endoscopic Surgeons. Dis Colon Rectum.

[2] Miettinen RP, Laitinen ST, Makela JT, Paakkonen ME (2000). Bowel preparation with oral polyethylene glycol electrolyte solution vs. no preparation in elective open colorectal surgery: prospective, randomized study. Dis Colon Rectum.

[3] Vo E, Massarweh NN, Chai CY, Tran Cao HS, Zamani N, Abraham S (2017). Clinical practice guidelines for enhanced recovery after colon and rectal surgery from the American Society of Colon and Rectal Surgeons and Society of American Gastrointestinal and Endoscopic Surgeons. JAMA Surg..

[4] Dolejs SC, Guzman MJ, Fajardo AD, Robb BW, Holcomb BK, Zarzaur BL (2017). Bowel preparation is associated with reduced morbidity in elderly patients undergoing elective colectomy. J Gastrointest Surg.

[5] Bucher P, Gervaz P, Soravia C, Mermillod B, Erne M, Morel P (2005). Randomized clinical trial of mechanical bowel preparation versus no preparation before elective left-sided colorectal surgery. Br J Surg.

[6] Shapira Z, Feldman L, Lavy R, Weissgarten J, Haitov Z, Halevy A (2010). Bowel preparation: comparing metabolic and electrolyte changes when using sodium phosphate/polyethylene glycol. Int J Surg.

[7] Zhu Y, Zhou W, Qi W, Liu W, Chen M, Zhu H (2017). Body mass index is a practical preoperative nutritional index for postoperative infectious complications after intestinal resection in patients with Crohn’s disease. Medicine (Baltimore).

[8] Toh JW, Stewart P, Rickard MJ, Leong R, Wang N, Young CJ (2016). Indications and surgical options for small bowel, large bowel and perianal Crohn’s disease. World J Gastroenterol.

[9] Feuerstein JD, Cheifetz AS (2017). Crohn disease: epidemiology, diagnosis, and management. Mayo Clin Proc.

[10] Garfinkle R, Abou-Khalil J, Morin N, Ghitulescu G, Vasilevsky CA, Gordon P (2017). Is there a role for oral antibiotic preparation alone before colorectal surgery? ACS-NSQIP analysis by coarsened exact matching. Dis Colon Rectum.

[11] Uchino M, Ikeuchi H, Bando T, Chohno T, Sasaki H, Horio Y (2017). Efficacy of preoperative oral antibiotic prophylaxis for the prevention of surgical site infections in patients with Crohn disease: a randomized controlled trial. Ann Surg.

[12] Kristo I, Stift A, Argeny S, Mittlbock M, Riss S (2016). Minimal-invasive approach for penetrating Crohn’s disease is not associated with increased complications. Surg Endosc.

[13] Riss S, Bittermann C, Zandl S, Kristo I, Stift A, Papay P (2010). Short-term complications of wide-lumen stapled anastomosis after ileocolic resection for Crohn’s disease: who is at risk?. Colorectal Dis.

[14] Dindo D, Demartines N, Clavien PA (2004). Classification of surgical complications: a new proposal with evaluation in a cohort of 6336 patients and results of a survey. Ann Surg.

[15] Horan TC, Gaynes RP, Martone WJ, Jarvis WR, Emori TG (1992). CDC definitions of nosocomial surgical site infections, 1992: a modification of CDC definitions of surgical wound infections. Am J Infect Control.

[16] von Elm E, Altman DG, Egger M, Pocock SJ, Gotzsche PC, Vandenbroucke JP (2007). The Strengthening the Reporting of Observational Studies in Epidemiology (STROBE) statement: guidelines for reporting observational studies. PLoS Med..

[17] Nichols RL, Broido P, Condon RE, Gorbach SL, Nyhus LM (1973). Effect of preoperative neomycin-erythromycin intestinal preparation on the incidence of infectious complications following colon surgery. Ann Surg.

[18] Zmora O, Wexner SD, Hajjar L, Park T, Efron JE, Nogueras JJ (2003). Trends in preparation for colorectal surgery: survey of the members of the American Society of Colon and Rectal Surgeons. Am Surg.

[19] Ohman KA, Wan L, Guthrie T, Johnston B, Leinicke JA, Glasgow SC (2017). Combination of oral antibiotics and mechanical bowel preparation reduces surgical site infection in colorectal surgery. J Am Coll Surg.

[20] Parthasarathy M, Greensmith M, Bowers D, Groot-Wassink T (2017). Risk factors for anastomotic leakage after colorectal resection: a retrospective analysis of 17 518 patients. Colorectal Dis.

[21] Klinger AL, Green H, Monlezun DJ, Beck D, Kann B, Vargas HD (2017). The role of bowel preparation in colorectal surgery: results of the 2012–2015 ACS-NSQIP data. Ann Surg.

[22] Kiran RP, Murray AC, Chiuzan C, Estrada D, Forde K (2015). Combined preoperative mechanical bowel preparation with oral antibiotics significantly reduces surgical site infection, anastomotic leak, and ileus after colorectal surgery. Ann Surg.

[23] Fa-Si-Oen P, Roumen R, Buitenweg J, van de Velde C, van Geldere D, Putter H (2005). Mechanical bowel preparation or not? Outcome of a multicenter, randomized trial in elective open colon surgery. Dis Colon Rectum.

[24] Shwaartz C, Fields AC, Sobrero M, Divino CM (2017). Does bowel preparation for inflammatory bowel disease surgery matter?. Colorectal Dis.

[25] Zangenberg MS, Horesh N, Kopylov U, El-Hussuna A (2017). Preoperative optimization of patients with inflammatory bowel disease undergoing gastrointestinal surgery: a systematic review. Int J Colorectal Dis.

[26] Iesalnieks I, Hoene M, Bittermann T, Schlitt HJ, Hackl C (2018). Mechanical Bowel Preparation (MBP) prior to elective colorectal resections in Crohn’s disease patients. Inflamm Bowel Dis.

[27] Oshima T, Takesue Y, Ikeuchi H, Matsuoka H, Nakajima K, Uchino M (2013). Preoperative oral antibiotics and intravenous antimicrobial prophylaxis reduce the incidence of surgical site infections in patients with ulcerative colitis undergoing IPAA. Dis Colon Rectum.

[28] Spinelli A, Bazzi P, Sacchi M, Danese S, Fiorino G, Malesci A (2013). Short-term outcomes of laparoscopy combined with enhanced recovery pathway after ileocecal resection for Crohn’s disease: a case-matched analysis. J Gastrointest Surg.

